# Supplementary material for: Mobile learning in dentistry: usage habits, attitudes and perceptions of undergraduate students
Source: PeerJ. 2019 Jul 29;7:e7391. doi: 10.7717/peerj.7391 (PMC6673424; doi:10.7717/peerj.7391)
Supplement: Supplemental Information 2 [file peerj-07-7391-s002.docx]

**Form^[[1]](#footnote-1)^**

Please select the most appropriate option of ‘5-Strongly Agree’, ‘4-Agree’, ‘3-Undecided’, ‘2-Disagree’, and ‘1-Strongly Disagree’ for the first 21 items of the questionnaire.

1. I think m-learning is the most suitable environment for students with different learning styles (visual, auditory, learning by doing and experiencing, etc.). …
2. I think that m-Learning can be more beneficial when it is combined with face to face learning in university courses. …
3. I think that M-Learning provides fast and practical learning. …
4. I agree that M-Learning provides permanent learning. …
5. I believe that it would be useful to spare my free time (Bus waiting, rest, etc.) with m-Learning outside of the course. …
6. I find it interesting m-learning since I don't want to carry books and course materials. …
7. I think m-Learning is a good opportunity to improve my research skills. …
8. Due to the potential dangers of the Internet (virus, etc.), I think m-Learning environment is unsafe. …
9. I think mobile devices are not suitable for use in m-Learning environment since they need to be charged regularly. …
10. I think m-Learning applications outside the classroom are useless because my attention is easily dispersed on the move. …
11. I do not find it appropriate to use mobile devices in the classroom because they are harmful to human health. …
12. I think M-Learning is not suitable for courses that require more reading and writing. …
13. I like to participate in m-Learning as I can access course materials faster. …
14. I believe that M-Learning supports planned and systematic study. …
15. I believe that mobile tools are useful for taking notes in class. …
16. I think mobile tools are useful for storing information. …
17. I believe that mobile devices with large screens are useful for m-learning. …
18. I think that M-Learning is an appropriate method for courses that require individual effort. …
19. I prefer to learn m-Learning because I can compensate myself for the lessons I have missed. …
20. I believe that M-Learning is useful for getting rapid feedback. …
21. I believe that using m-Learning in the courses at the University will increase my freedom of learning. …
22. Gender

Female □ Male □

1. Year of Birth: …
2. Please tick what you have from the following technologies?
3. Smart phone: Present □ Absent □
4. Tablet: Present □ Absent □
5. Laptop: Present □ Absent □
6. Other: …
7. Do you have an internet package on your phone?

Yes … (GB) □ No □

1. Indicate your habits for mobile devices.
2. I carry a charger with me. Yes □ No □
3. I have a portable power supply with me. Yes □ No □
4. I check my phone as soon as I wake up. Yes □ No □
5. I check my phone before I go to sleep. Yes □ No □
6. Choose where you use the Internet
7. I don't have internet access □
8. Home □
9. Dorm □
10. University □
11. Mobile phone □
12. Cafe-Restaurant □
13. Free public wifi (Public Wifi) □
14. Other: …
15. Write down your average daily internet usage time: …
16. Sort the following options from the internet for you to use more often.
17. Online shopping □
18. Connecting to Social Networks □
19. Watching video □
20. Reading scientific articles □
21. Accessing course materials □
22. Checking e-mails □
23. Reading news □
24. Communicating □
25. Listening to music □
26. Playing games □
27. The use of mobile tools in the courses given in dentistry is beneficial (please select only one option)
28. Strongly Disagreed □
29. Disagreed □
30. Undecided □
31. Agreed □
32. Strongly Agreed □
33. Are there any mobile phone applications that you use for training purposes? Please indicate if applicable.

|  |
| --- |

1. What are the positive aspects of course learning with mobile learning?

|  |
| --- |

1. What are the negative aspects of course learning with mobile learning?

|  |
| --- |

1. What are your expectations for the use of mobile tools in the processing of courses?

|  |
| --- |

1. What kind of content do you want to see on mobile devices to increase your success in your courses?

|  |
| --- |

1. English Translation of Turkish Questionnaire [↑](#footnote-ref-1)
